# Diverse Reductive Dehalogenases Are Associated with Clostridiales-Enriched Microcosms Dechlorinating 1,2-Dichloroethane

**DOI:** 10.1155/2015/242856

**Published:** 2015-07-26

**Authors:** Giuseppe Merlino, Annalisa Balloi, Massimo Marzorati, Francesca Mapelli, Aurora Rizzi, Davide Lavazza, Francesca de Ferra, Giovanna Carpani, Daniele Daffonchio

**Affiliations:** ^1^Department of Food, Environmental and Nutritional Sciences (DeFENS), University of Milan, 20133 Milan, Italy; ^2^Biological and Environmental Sciences and Engineering Division, King Abdullah University of Science and Technology, Thuwal 23955-6900, Saudi Arabia; ^3^Laboratory for Microbial Ecology and Technology (LabMET), Ghent University, 9000 Ghent, Belgium; ^4^Research Center for Non-Conventional Energy, Istituto Eni Donegani, Environmental Technologies, 20097 San Donato Milanese, Italy

## Abstract

The achievement of successful biostimulation of active microbiomes for the cleanup of a polluted site is strictly dependent on the knowledge of the key microorganisms equipped with the relevant catabolic genes responsible for the degradation process. In this work, we present the characterization of the bacterial community developed in anaerobic microcosms after biostimulation with the electron donor lactate of groundwater polluted with 1,2-dichloroethane (1,2-DCA). Through a multilevel analysis, we have assessed (i) the structural analysis of the bacterial community; (ii) the identification of putative dehalorespiring bacteria; (iii) the characterization of functional genes encoding for putative 1,2-DCA reductive dehalogenases (RDs). Following the biostimulation treatment, the structure of the bacterial community underwent a notable change of the main phylotypes, with the enrichment of representatives of the order *Clostridiales*. Through PCR targeting conserved regions within known RD genes, four novel variants of RDs previously associated with the reductive dechlorination of 1,2-DCA were identified in the metagenome of the Clostridiales-dominated bacterial community.

## 1. Introduction

Chlorinated compounds are among the major global environmental contaminants [1]. A large number of compounds of this class of chemicals have been produced in big quantities for several applications in industry and agriculture such as biocides, flame retardants, solvents, and intermediates for the production of polymers (e.g., PVC) [1, 2]. Their widespread diffusion and use resulted in the massive release in the environment, with consequent concerns for human health due to the persistence, tendency to bioaccumulate, and proven toxicity [2, 3]. Due to the physicochemical properties, most halogenated compounds are recalcitrant to aerobic dehalogenation and tend to accumulate in anoxic ecosystems (e.g., soils and groundwater aquifers). For this reason, many of the research efforts of the last decades, aimed at defining efficient remediation approaches, were focused on the investigation of anaerobic degrading potential of microbial cultures enriched/isolated from typical anoxic environments. Chlorinated solvents in these conditions can undergo biologically mediated degradation through either oxidative, fermentative, or reductive processes [4]. Particular interest has been focused on the third kind of biodegradation process, since several studies have highlighted the high dechlorinating performances of pure and mixed microbial cultures through reductive dehalogenation [5–10]. The peculiarity of this process is that the chlorinated molecule is the terminal electron acceptor of the membrane-bound electron transport chain coupled to the generation of energy in the form of ATP [4].

Among the wide variety of chlorinated solvents, 1,2-dichloroethane (1,2-DCA) is considered one of the major pollutants, being one of the most widespread contaminating groundwater worldwide and being classified as a possible human carcinogenic agent by many environmental agencies [2]. 1,2-DCA can undergo either partial or complete detoxification in anoxic conditions through three different mechanisms: dichloroelimination, reductive hydrogenolysis, and dehydrochlorination [5]. Among these, only the first mechanism leads to the production of the harmless end-product ethylene, while the other two generate molecules whose toxicity is even higher than 1,2-DCA, in particular the carcinogenic vinyl chloride (VC). Key enzymes involved in this anaerobic dehalogenating metabolism are the reductive dehalogenases (RDs), a class of cobalamin-dependent oxygen-sensitive enzymes, usually associated with the membranes and capable of replacing halogen atoms with hydrogen ones from the carbon backbone of the molecules [4, 11]. Different studies have unveiled details about structure and function of some enzymes belonging to this class [12–14]. Only recently, novel RDs sequences were correlated with 1,2-DCA dechlorination to ethene in a 1,2-DCA dehalogenating enrichment culture containing a* Dehalobacter* sp. WL (rdhA1, rdhA2, and rdhA3) [15] and* in situ* in the upper water layer of a double layer aquifer contaminated by 1,2-DCA (RD54) [16]. The enrichment culture setup from the upper layer of the aquifer (culture 6VS) contained both* Dehalobacter* and* Desulfitobacterium* spp. In addition to the two just cited representatives of the phylum Firmicutes, only few other bacterial strains have been identified so far as capable of detoxifying 1,2-DCA to ethylene via dichloroelimination. Papers [17, 18] were the first to report the ability of two* Chloroflexi* representatives, respectively,* Dehalococcoides ethenogenes* strain 195 and* Dehalococcoides *sp. strain BAV1 to grow on 1,2-DCA as electron acceptor producing ethylene as the main end product. A peculiarity of the species of this genus is their capability to grow exclusively on chlorinated compounds as electron acceptor. Other representatives of the phylum Chloroflexi with the ability to grow on 1,2-DCA described recently are two strains of the genus* Dehalogenimonas*:* D. lykanthroporepellens* [19] and* D. alkenigignens* [20], both characterized by the ability to degrade high concentration of 1,2-DCA up to 8.7 mM [21].

In the present work, the dechlorinating bacterial microbiome in the lower layer of the same aquifer investigated by [16] has been characterized in terms of structure and functionality, before and after the supplement with lactate. We have investigated (i) the response of the indigenous microbial community to lactate treatment, (ii) the key microbial dehalogenating bacteria, and (iii) the RDs involved in the dehalogenation process.

## 2. Materials and Methods

### 2.1. Preparation of Enrichment Cultures

Evaluation of biodegradation of 1,2-DCA was carried out in anaerobic microcosms set-up with groundwater collected from the lower layer (from 14 m to 40 m deep) of an aquifer previously studied in northern Italy [7, 9, 16], heavily polluted exclusively by 1,2-DCA more than 30 years ago. Concentration of the contaminant in the lower aquifer was about 197 ± 23 mg L^−1^ and it was maintained the same during preparation of anaerobic cultures. The other chlorinated ethane and ethene were not detected. Thirty mL triplicate microcosms were assembled in 50 mL vials under an atmosphere of 80% N_2_, 15% CO_2_, and 5% H_2_ in the anaerobic glove-box Simplicity 888 (Plas-Labs, USA). Culturing medium consisted of a 1 : 200 dilution of a trace elements solution (12.8 g L^−1^ nitrilotriacetic acid, 1.35 g L^−1^ FeCl_3_·6 H_2_O, 0.1 g L^−1^ MnCl_2_·4 H_2_O, 0.024 g L^−1^ CoCl_2_·6 H_2_O, 0.1 g L^−1^ CaCl_2_·2 H_2_O, 0.1 g L^−1^ ZnCl_2_, 0.025 g L^−1^ CuCl_2_·2 H_2_O, 0.01 g L^−1^ H_3_BO_3_, 0.024 g L^−1^ Na_2_MoO_4_·2 H_2_O, 1 g L^−1^ NaCl, 0.12 g L^−1^ NiCl_2_·6 H_2_O, and 0.026 g L^−1^ Na_2_SeO_3_·5 H_2_O), a supplementary salt solution (43 mg L^−1^ NH_4_Cl, 0.5 g L^−1^ KH_2_PO_4_, 0.2 g L^−1^ MgCl_2_·6 H_2_O, and 0.01 g L^−1^ CaCl_2_·2 H_2_O), 0.05% (w/v) yeast extract, 0.5 mM 4-(2-hydroxyethyl) piperazine-1-ethanesulfonic acid/NaOH (Hepes/NaOH) solution pH 7.0, cysteine 1 mM, and vitamin B_12_ 50 mg L^−1^. Lactate at final concentration of 5 mM was used as the only carbon source and electron donor [22]. Control microcosms were prepared by incubating parallel vials containing the same culturing medium with filter-sterilized groundwater samples. All microcosms were sealed with teflon-faced septa and aluminum crimp seals and statically incubated in the dark at 23°C.

Concentration of 1,2-DCA and of its possible degradation products, ethane and VC, was evaluated by the injection of 500 *μ*L samples of headspace of the microcosms in a Gas Chromatograph/Flame Ionization Detector (GC/FID) Agilent 7694 equipped with a DB624 column (J&W Scientific, Folsom, CA). The temperature of the oven and of the detector was set at 80 and 200°C, respectively. 1,2-DCA limit of detection was 1.0 *μ*g L^−1^.

### 2.2. Genomic DNA Isolation

Groundwater and microcosm samples, respectively, 30 and 1.5 mL (samples withdrawn from replicate cultures were pooled together for a total final volume of 4.5 mL), were filtered using Sterivex filters (Millipore, Milan, Italy). Total genomic DNA was extracted from the filtered bacterial cells by incubating the filter with 2 mL of a lysis solution containing 1 mg mL^−1^ lysozyme, 1% (w/v) sodium dodecyl sulphate, and 0.5 mg mL^−1^ proteinase K and purified as previously described by Murray et al. [23].

### 2.3. PCR Amplification of Bacterial and Archaeal* 16S rRNA* and RD Genes

Bacterial* 16S rRNA* gene was amplified from the groundwater metagenome using universal primers 27f and 1492r [24] with the following reaction concentrations in a final volume of 50 *μ*L: 1X PCR buffer, 1.5 mM MgCl_2_, 0.12 mM dNTPs, 0.3 *μ*M of each primer, and 1 U of Taq polymerase. Thermal protocol used was the following: initial denaturation at 94°C for 5 minutes, followed by 5 cycles consisting of denaturation at 94°C for 1 minute, annealing at 50°C for 1 minute, and extension at 72°C for 2 minutes and subsequently by 30 cycles consisting of denaturation at 94°C for 1 minute, annealing at 55°C for 1 minute, and extension at 72°C for 2 minutes. A final extension at 72°C for 10 minutes was performed.

PCR with specific primers for Archaea was attempted in order to investigate the* 16S rRNA* diversity of this group of prokaryotes. A first step was carried out using universal archaeal forward primers 21f and 1492r, using the same reaction mix and thermal protocol presented elsewhere [25]. Since the first PCR step did not give any amplicon, a second round of PCR using primers PARCH 340F and 934R was attempted, as previously described by Cytryn et al. [26]. However, also this second amplification attempt did not result in any PCR product.

A 2000 bp region of the reductive dehalogenase gene cluster previously identified by Marzorati and colleagues [16] was amplified using primers PceAFor1 (5′-ACGT GCA ATT ATT ATT AAG G-3′) and DcaBRev (5′-TGG TAT TCA CGC TCC GA-3′), in order to construct a gene library of the functional genes encoding for the RD specific for 1,2-DCA degradation. The reaction mix was prepared as follows: 1X PCR buffer, 1.5 mM MgCl_2_, 0.2 mM dNTPs, 0.6 *μ*M of each primer, and 1 U of Taq polymerase in a final volume of 25 *μ*L. The thermal consisted of an initial denaturation at 94°C for 3 minutes, followed by 31 cycles of denaturation at 94°C for 30 seconds, annealing at 54°C for 1 minute, extension at 72°C for 2 minutes, and subsequently a final extension at 72°C for 7 minutes.

### 2.4. *16S rRNA* and RD Genes Libraries

Cloning reactions were performed with pGEM cloning kit (pGEM-T Easy Vector Systems, Promega, Milan, Italy) following the instructions of the manufacturer. Sixty ng of PCR product was used for each cloning reaction, maintaining a molar ratio insert : vector of 3 : 1. A PCR assay was performed on white positive colonies to amplify the insert using primers T7 (3′-CTA ATA CGA CTC ACT ATA GGG-5′) and SP6 (3′-ATT TAG GTG ACA CTA TAG AAT A-5′). PCR products were purified with QIAquick PCR Purification Kit (Qiagen, Milan, Italy) according to the manufacturer's instructions.

### 2.5. *16S rRNA* Gene Phylogenetic and RDs Diversity Analyses

Clones from bacterial* 16S rRNA* and RD genes libraries were sequenced, respectively, with primers 27F and PceAFor1, using the ABI Prism BigDye terminator cycle sequencing kit (Applied Biosystems, Milan, Italy) and an ABI 310 automated sequencer (Applied Biosystems). Sequences were edited with software Chromas Lite version 2.01. Sequences of the* 16S rRNA* bacterial libraries were checked for chimeric PCR products using DECIPHER online software tool [27] and nonchimeric sequences were then used to define operational taxonomic units (OTUs) at 99% of similarity (OTU99) using DOTUR [28]. Shannon diversity index (*H*′) was calculated using software PAST version 3.02 [29]. The sequences of the OTU representatives were analysed using the Basic Local Alignment Search Tool (BLAST) of the online GenBank database [30] and by the CLASSIFIER Match Tool version 2.6 of Ribosomal Database Project II (RDP II) [31]. Pareto-Lorenz distribution curves (PL curves) [32, 33] were constructed based on the* 16S rRNA* gene clone library results, in order to graphically evaluate the community organization (Co) of the bacterial consortia as described elsewhere [34].

Identification of the closest relative match for the RDs libraries was carried out comparing the sequences with BLAST. Sequences of functional gene libraries were used to construct neighbour-joining phylogenetic tree, with bootstrap of 1000 repetitions, and compute the evolutionary distances through Kimura's two-parameter model using software MEGA version 5 [35]. Alignment of amino acids sequences of the functional genes deducted from the nucleotide sequences of the RDs libraries was carried as described elsewhere [36] in order to identify characteristic amino acid residues conserved in all RDs.

### 2.6. Nucleotide Sequence Accession Numbers

Nucleotide sequences of all clones identified in this study were deposited in the EMBL nucleotide sequence database (GenBank/EMBL/DDBJ) under the accession numbers FM210335, FM204948 to FM204979 for bacterial* 16S rRNA* genes, and FM204931 to FM204934 for RDs sequences.

## 3. Results and Discussion

### 3.1. Structure and Diversity of the Bacterial Community before and after the Biostimulation

A triplicate series of anaerobic microcosms with a concentration of 1,2-DCA of 197 ± 23 mg L^−1^ was set up using groundwater from the lower layer of a double aquifer contaminated by 1,2-DCA analogously to the experiments previously run for the upper layer of the same aquifer system [9]. Following the addition of 5 mM lactate, all the microcosms readily degraded 1,2-DCA in 15 days, with an average dechlorination rate of 13.1 ± 1.9 mg L^−1^ day^−1^. Ethane accumulated as the only end product while the toxic intermediate VC was always below the detection limit, suggesting that degradation of 1,2-DCA occurred only via dichloroelimination [22]. The analogous biostimulation treatment with groundwater from the upper layer [9] gave considerably higher degradation rate of 69.4 ± 2.2 mg L^−1^ day^−1^. It can be speculated that this almost-four times statistically significant difference (as determined by Student's *t*-test with *P* < 0.000001) between the two layers was possibly due to differences in the enriched dechlorinating species.

The bacterial diversity of the community before (*t*_0_) and after (*t*_1_) the biostimulation treatment was evaluated by establishing* 16S rRNA* gene clone libraries. Differently from what was observed previously on the upper layer of the aquifer [9], PCR with specific primers for Archaea did not result in any amplicon either before or after lactate amendment, even after a second round of PCR using nested primers. This suggests that in the lower aquifer Archaea are not implicated in the dechlorination process.

**Figure 1:**
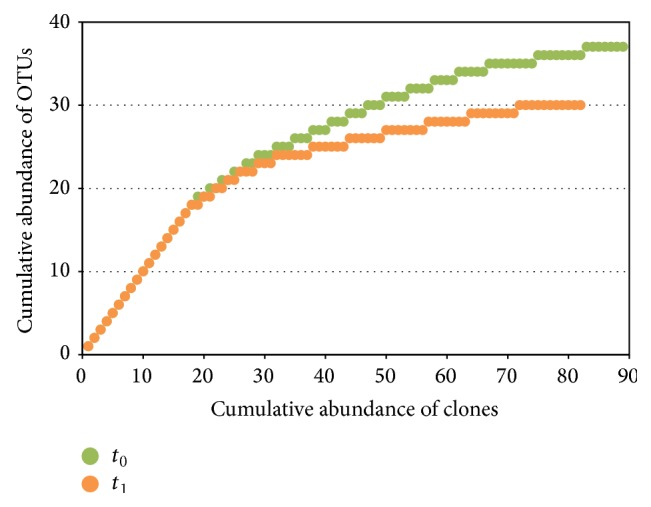
Rarefaction curves calculated for the bacterial* 16S rRNA* gene clone libraries, before (*t*_0_) and after (*t*_1_) the biostimulation treatment with lactate.

The bacterial libraries were made of 91 clones each. Chimera check allowed excluding 6.0% of all the sequences obtained, lowering the number of clones to 89 and 82 for *t*_0_ and *t*_1_, respectively. Good coverage of the dominant OTUs was confirmed with rarefaction analysis of the clone libraries (Figure 1). The diversity of the bacterial communities was evaluated by means of two parameters: (i) Shannon index (*H*′), which allowed describing the species richness, and (ii) evenness index, used to describe the relative abundance among species within the communities. Shannon index, which accounts for both abundance and evenness of the species present, was 3.33 in the lower aquifer with respect to 1.91 in the upper one, indicating that the lower aquifer hosted greater species diversity than the upper one before the treatment. At *t*_1_ after lactate amendment the Shannon index in the lower aquifer decreased (2.88 versus 3.33), while in the upper aquifer it remained almost unchanged (1.81 versus 1.91). The small *H*′ variation in the lower aquifer suggests that relatively limited changes in the biodiversity of the bacterial community occurred after the biostimulation treatment.

**Figure 2:**
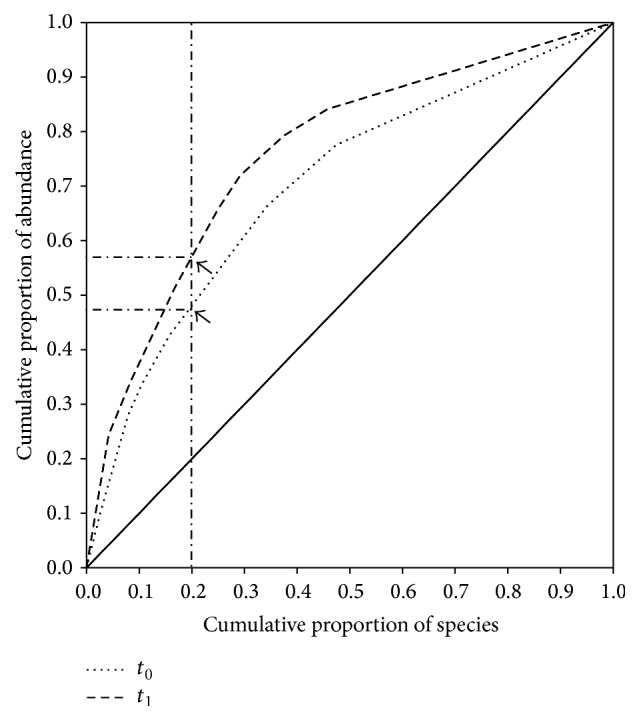
Pareto-Lorenz distribution curves representation of the community organization (Co) of the microbial communities before (*t*_0_, dotted line) and after (*t*_1_, dashed line) the treatment with lactate. The continuous line represents the perfect evenness. Black arrows indicate the OTU cumulative proportion of abundances corresponding to an OTU cumulative proportion of 20%.

The PL curves, used as a graphical estimator of the Co [32, 33], confirmed the little bacterial diversity change in the lower aquifer, in response to the biostimulation treatment (Figure 2). Co curves at *t*_0_ showed a situation where 20% of the OTUs represented about 48% of the total abundance of clones. After the lactate treatment, this proportion grew to 58%, indicating that both communities were characterized by a relatively moderate organization. It can be speculated that the bacterial community of the lower aquifer was characterized by a slight dominance both before and after the biostimulation treatment and sudden changes in the environmental conditions, as those determined by the supplement of lactate, would change the dominant species but would not influence the overall Co and evenness structure of the community.

**Figure 3:**
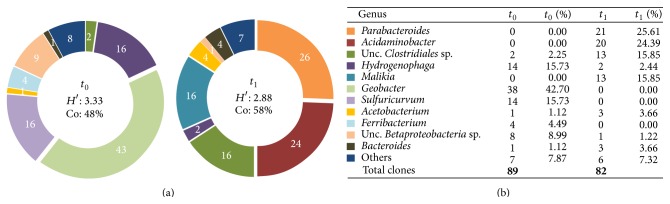
(a) Pie charts illustrating the percentages of clones, identified in the bacterial communities at *t*_0_ and at *t*_1_, grouped in phylotypes at genus level; *H*′: Shannon Index and Co: community organization (evenness index); (b) table showing the abundance and the percentages of clones grouped in phylotypes at genus level.

The 171 clones obtained in the two libraries were grouped in 60 distinct OTUs. A summary of the representatives of each OTU identified through BLAST and CLASSIFIER is presented in Table 1, together with the number of clones of each OTU occurring before and after the biostimulation treatment. Thirty-eight of the 60 OTUs were detected before the lactate amendment and 24 after it, with only two OTUs detected both at *t*_0_ and at *t*_1_, respectively, affiliated to uncultured Clostridiales and to* Sulfuricurvum *sp. The bacterial community at *t*_0_ was characterized by a wider diversity, with dominating sequences belonging to Proteobacteria phylum (Table 1, Figure 3): in order of abundance *δ*- (38 clones describing 15 OTUs), *β-* (26 clones describing 11 OTUs), and *ε-*Proteobacteria (15 clones describing 4 OTUs). Within the *δ-*Proteobacteria, all the sequences were closely related to genus* Geobacter* (97–100% identity), the majority of which were affiliated to uncultured* Geobacter *sp. and* Geobacter thiogenes* (15 clones each). Species of the genus* Geobacter* were commonly found in freshwater sediments and subsurface environments [37]. Previously, de Wever and colleagues [38] described the ability of* Geobacter thiogenes* to dechlorinate trichloroacetic acid. Another representative of the* Geobacter* clade,* G. lovleyi* (6 clones), a known tetrachloroethene-dechlorinating bacterium [39], was also identified. Within the *β-* and *ε-*Proteobacteria groups, the most represented phylotypes were closely related to* Hydrogenophaga taeniospiralis* (11 clones) and* Sulfuricurvum kujiense* (10 clones). These two genera are environmental microorganisms typically detected in contaminated freshwater ecosystems [40]. For instance,* H. pseudoflava* was identified by Liang and colleagues [41] in a TCE-degrading consortium enriched from TCE-contaminated aquifer sediments and groundwater. A psychrotrophic* H. pseudoflava* strain IA3-A was isolated from polychlorinated biphenyls-contaminated soil and grew on biphenyl as sole carbon and energy source [42]. Both genera,* Hydrogenophaga* and* Sulfuricurvum*, were recently enriched and associated with NO_3_^−^-reduction in a membrane biofilm reactor inoculated with wastewater sludge and treating perchlorate [43].

The biostimulation with lactate determined a remarkable change of the diversity within the bacterial community. A lower diversity (24 OTUs) was observed and phylotypes related to Firmicutes, Bacteroidetes, and *β-*Proteobacteria, not detected at *t*_0_, became dominant; that is, representatives of genera* Acidaminobacter* (20 clones),* Parabacteroides* (21 clones), and* Malikia* (13 clones) were strongly enriched (Table 1, Figure 3). Conversely,* Geobacter*,* Hydrogenophaga*, and* Sulfuricurvum*, the phylotypes dominating the consortium before the treatment, were not detected in the library after the treatment. A similar shift of diversity was previously observed in the upper layer microcosms [9]. However, while in the upper layer of the aquifer phylotypes of known 1,2-DCA dehalogenating genera of the Clostridiales (*Desulfitobacterium* and* Dehalobacter*) were enriched after the biostimulation with lactate, none of the genera enriched in microcosms from the lower layer has been so far associated with reductive dechlorination of 1,2-DCA. Among the phylotypes enriched in the lower layer microcosms, the only characterized representative of genus* Acidaminobacter*,* A. hydrogenoformans*, has been described as a fermentative species whose growth is enhanced by cocultivation with a hydrogen-consuming partner; for example, in our study, it could be a microbe able to couple the H_2_ consumption with 1,2-DCA reductive dechlorination [44]. Interestingly, another phylotype enriched at *t*_1_ was related to an uncultured Clostridiales bacterium (12 clones) and, noteworthily, the only reductive dehalogenases specific for 1,2-DCA identified so far were previously associated only with 2 genera belonging to Clostridiales order:* Desulfitobacterium* [16] and* Dehalobacter* [15]. Taken together, these data indicate that in the lower aquifer the lactate amendment enriched different phylogenetically distant taxa previously not associated with 1,2-DCA dechlorination, suggesting that novel reductive dechlorinators may mediate such a process.

### 3.2. Reductive Dehalogenase Gene Libraries

The reductive dehalogenase diversity in the lower aquifer was investigated in response to lactate biostimulation to evaluate whether reductive dehalogenating functional redundancy could be associated with the diversity pattern depicted by the* 16S rRNA* gene libraries. In previous works, a complete sequence of one RD gene cluster specifically adapted to 1,2-DCA was obtained from microcosms of the upper layer of the aquifer [16]. Three genes (*dcaB*,* dcaC*, and* dcaT*) of the identified RD cluster presented high nucleotide identity (above 98%) with the RDs specific for chlorinated alkenes, but the gene coding for the main catalytic subunit of the reductive dehalogenase (*dcaA*) presented only 94% and 90% nucleotide and amino acid identities. The sequence differences were associated with dechlorination of 1,2-DCA since* Desulfitobacterium dichloroeliminans* strain DCA1, capable of dechlorinating 1,2-DCA but not chlorinated ethene, showed the same amino acid signatures in the two sole RDs identified in the genome [16].

**Figure 4:**
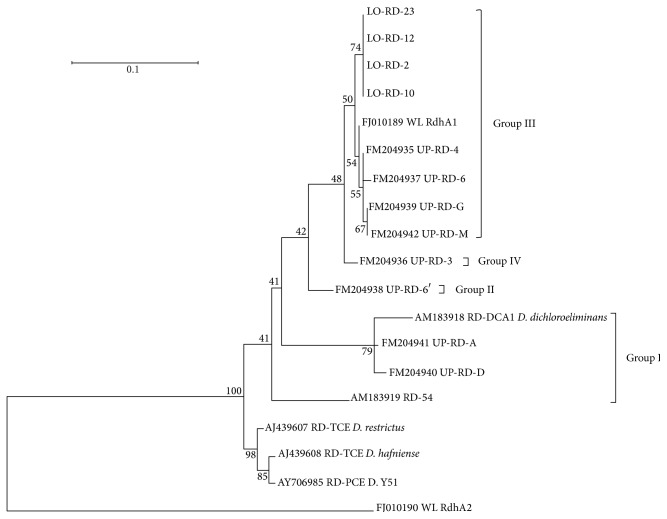
Neighbour-joining tree with branch length to assess the relationship between DcaA of the new RDs identified in the lower aquifer (LO-RD-X) and those previously characterized from the upper aquifer (RD-54 [16] and UP-RD-X [9]) and from* D. dichloroeliminans* strain DCA1 (RD-DCA1 [16]). Other A subunits of PceA of* Dehalobacter restrictus* strain DSMZ 9455T (RD-TCE* D*.* restrictus*: AJ439607),* Desulfitobacterium hafniense* strain TCE1 (RD-TCE* D. hafniense*: AJ439608),* Desulfitobacterium *sp. strain Y51 (RD-PCE D. Y51: AY706985), WL rdhA1 (FJ010189), and WL rdhA2 (FJ010190) are also reported. The numbers at each branch point represent percentage of bootstrap calculated from 1000 replicate trees. The scale bar represents the sequence divergence.

**Figure 5:**
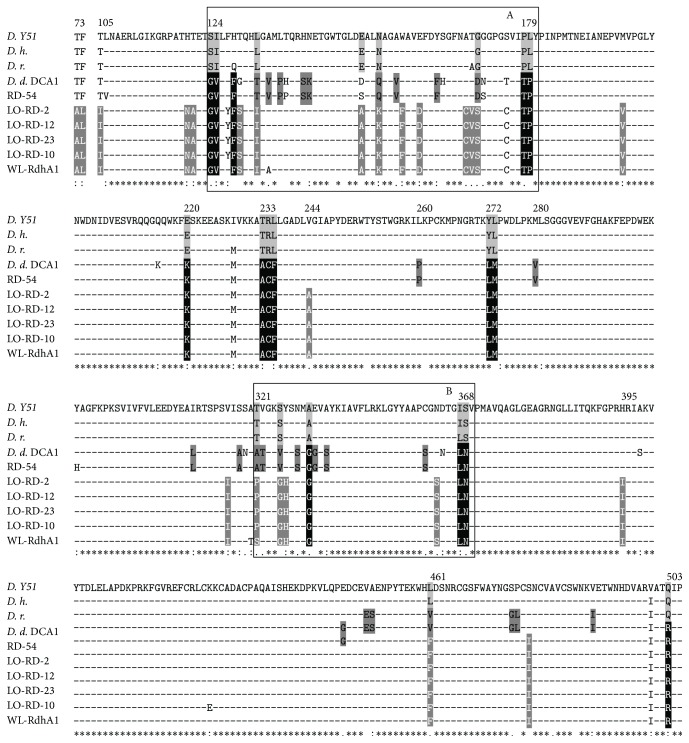
Amino acid alignment of the DcaA proteins of the new identified RDs, with those previously identified in the groundwater (RD-54: AM183919) and in* D. dichloroeliminans* strain DCA1 (*D. d.* DCA1: AM183918), with PceA of* Desulfitobacterium *sp. strain Y51 (*D*. Y51: AY706985),* D. hafniense* strain TCE1 (*D. h.*: AJ439608), and* D. restrictus* strain DSMZ 9455T (*D. r.*: AJ439607) and with the WL rdhA1 (FJ010189). Black line (blocks A and B) rectangles indicate two amino acid stretches where 53% of the total amino acid diversity resides between DcaA and PceA [16]. Within blocks A and B of the selected RDs sequences, as well in other smaller regions of the DcaA subunit, it was possible to identify amino acids specific for (i) PceA of the PCE-RDs (black residues in a light gray background), (ii) DcaA of group I, specific for WL rdhA1 and for the reductive dehalogenases identified in the low aquifer after the lactate treatment (white residues in a light gray background), (iii) those of group II proposed to be specific for 1,2-DCA RDs from* Desulfitobacterium* (black residues in a dark gray background), (iv) and finally those common to all the RDs within groups I and II but not conserved in the PCE-specific RDs (white residues in a black background). Asterisks, colons, and dots below the alignment indicate an identical position in all the proteins, a position with a conservative substitution, and a position with a semiconservative substitution, respectively.

Using the same RD-targeting PCR approach of Marzorati et al. [16], a total of 17 clones were obtained after the treatment, representing four different RDs.  Figure 4 shows their phylogenetic relationship with known RDs. The RD sequences found in the lower aquifer layer were grouped in one cluster together with those previously identified in the upper aquifer layer [9]. The percentage of similarity among the newly identified RDs was between 100 and 99% and shared 99% nt identity with WL rdhA1, one of the three RDs identified by Grostern and Edwards [15], in a 1,2-DCA degrading coculture where the main representative was* Dehalobacter *sp. WL. It has been previously shown that the 53% of the total amino acid diversity of* dcaA* RDs (RD-54 and RD-DCA1) with respect to* pceA* RDs specific for tetrachloroethene (PCE; RDs from* Dehalobacter restrictus* strain DSMZ 9455T,* Desulfitobacterium *sp. strain Y51, and* Desulfitobacterium hafniense* strain PCE-S) [12, 45, 46] was mainly localized in two small regions (blocks A and B, Figure 5) that represent only 19% (104 amino acids over 551) of the total* dcaA* residues. These two regions of hypervariability were proposed to be involved in the recognition of 1,2-DCA or in general in the substrate specificity of RDs [16]. The alignment of the RDs identified in the lower aquifer layer with the above-indicated homologs was possible to identify the two mentioned hypervariable regions overlapping with blocks A and B (Figure 5). The alignment permitted identifying amino acids specifically associated with (i) PceA of the PCE-RDs (black residues in a light grey background); (ii) DcaA of group I, specific for WL rdhA1 and for the reductive dehalogenases enriched from the lower aquifer layer (white residues in a light grey background); (iii) DcaA of group II proposed to be specific for 1,2-DCA RDs from* Desulfitobacterium* (black residues in a dark grey background); (iv) all the RDs within groups I and II but not conserved in the PCE-specific RDs (white residues in a black background).

## 4. Conclusions

By comparing the diversity of bacteria and RDs in the two aquifer layers following biostimulation with lactate, it can be argued that the RDs linked to 1,2-DCA reductive dechlorination, despite being diverse, are structurally conserved. However, they can be associated with different bacterial carriers selected by the environmental conditions of the specific aquifer, indicating their plasticity to adapt to different cellular scaffolds and machineries.

## Figures and Tables

**Table 1:** Summary of the OTU representatives, identified in the 16S rRNA gene libraries.

OTU	Clones		Basic Local Alignment Search Tool-GenBank			CLASSIFIER Match Tool-Ribosomal Database Project II		
	*t*_0_	*t*_1_	Closest described relative	Acc. *n*°	% identity	Phylogenetic group	Closest classified relative	% certainty^A^
**1**	3	0	*Geobacter thiogenes *	NR_028775	98.96	Deltaproteobacteria	*Geobacter *	100
**2**	3	0	*Geobacter thiogenes *	NR_028775	98.78	Deltaproteobacteria	*Geobacter *	100
**3**	8	0	*Geobacter thiogenes *	NR_028775	99.09	Deltaproteobacteria	*Geobacter *	100
**4**	3	0	Unc. bacterium	AM410013	97.34	Deltaproteobacteria	*Geobacter *	100
**5**	1	0	Unc. *Geobacter* sp.	FM204959	98.63	Deltaproteobacteria	*Geobacter *	100
**6**	1	0	Unc. *Geobacter* sp.	EU266833	98.62	Deltaproteobacteria	*Geobacter *	100
**7**	3	0	Unc. *Geobacter* sp.	AY752765	98.56	Deltaproteobacteria	*Geobacter *	100
**8**	1	0	Unc. *Geobacter* sp.	AY752765	98.42	Deltaproteobacteria	*Geobacter *	100
**9**	1	0	Unc. *Dehalobacter* sp.	HM748813	99.43	Clostridia	*Acetobacterium *	100
**10**	4	0	Unc. *Sulfurimonas* sp.	KF851122	98.34	Epsilonproteobacteria	*Sulfuricurvum *	100
**11**	4	0	*Ferribacterium* sp. *7A-631 *	KF441656	99.75	Betaproteobacteria	*Ferribacterium *	93
**12**	1	0	Unc. Gallionellaceae bacterium	EU266776	96.48	Proteobacteria	Betaproteobacteria	100
**13**	1	0	Unc. Rhodocyclaceae bacterium	JQ279024	98.83	Betaproteobacteria	Rhodocyclaceae	98
**14**	1	0	Unc. Rhodocyclaceae bacterium	HQ003471	97.64	Betaproteobacteria	Rhodocyclaceae	100
**15**	0	1	*Acinetobacter baumannii *	KJ958271	99.60	Gammaproteobacteria	*Acinetobacter *	100
**16**	0	1	*Pseudomonas putida *	GU396283	98.97	Gammaproteobacteria	*Pseudomonas *	100
**17**	0	7	Unc. *Bacteroides* sp.	AB529592	99.44	Bacteroidia	*Parabacteroides *	99
**18**	0	1	Unc. Bacteroidetes bacterium	FJ535139	98.31	Bacteroidia	*Parabacteroides *	100
**19**	0	1	Unc. *Bacteroides* sp.	JQ624314	99.75	Bacteroidia	*Parabacteroides *	100
**20**	0	1	Unc. Bacteroidetes bacterium	DQ676360	98.97	Bacteroidia	Porphyromonadaceae	99
**21**	0	1	Unc. *Bacteroides* sp.	FM204969	99.88	Bacteroidia	*Parabacteroides *	99
**22**	0	1	Unc. *Acidaminobacter* sp.	HM217344	98.61	Clostridia	Clostridiales Incertae Sedis XII	91
**23**	0	6	Unc. *Acidaminobacter* sp.	HM217344	98.78	Clostridia	Clostridiales	100
**24**	0	5	Unc. *Acidaminobacter* sp.	HM217344	99.46	Clostridia	Clostridiales Incertae Sedis XII	80
**25**	2	0	Unc. *Hydrogenophaga* sp.	HM124825	99.67	Betaproteobacteria	*Hydrogenophaga *	100
**26**	3	0	*Hydrogenophaga taeniospiralis *	AY771764	98.02	Betaproteobacteria	*Hydrogenophaga *	100
**27**	0	7	*Malikia spinosa *	NR_040904	99.86	Betaproteobacteria	*Malikia *	100
**28**	1	0	Unc. *Hydrogenophaga* sp.	DQ413154	98.70	Betaproteobacteria	*Hydrogenophaga *	100
**29**	1	0	Unc. Elusimicrobia bacterium	GU236016	94.55	Elusimicrobia	*Elusimicrobium *	98
**30**	0	2	*Hydrogenophaga taeniospiralis *	AY771764	98.75	Betaproteobacteria	*Hydrogenophaga *	95
**31**	8	0	*Hydrogenophaga taeniospiralis *	AY771764	99.06	Betaproteobacteria	*Hydrogenophaga *	98
**32**	0	6	*Malikia spinosa *	NR_040904	99.73	Betaproteobacteria	*Malikia *	88
**33**	1	0	Unc. *Acidovorax* sp.	AM084039	99.04	Betaproteobacteria	Comamonadaceae	100
**34**	3	1	Unc. *Dechloromonas* sp.	JN679130	98.95	Betaproteobacteria	Rhodocyclaceae	100
**35**	1	0	Unc. *Gallionella* sp.	FJ391502	98.72	Proteobacteria	Betaproteobacteria	100
**36**	0	2	*Vogesella indigofera *	NR_040800	99.60	Betaproteobacteria	*Vogesella *	100
**37**	0	1	*Shewanella putrefaciens *	JN019028	99.87	Gammaproteobacteria	*Shewanella *	100
**38**	9	0	*Sulfuricurvum kujiense *	CP002355	99.22	Epsilonproteobacteria	*Sulfuricurvum *	100
**39**	1	0	Unc. *Arcobacter* sp.	JQ861849	97.96	Epsilonproteobacteria	*Arcobacter *	93
**40**	2	0	*Geobacter metallireducens *	NR_075011	98.31	Deltaproteobacteria	*Geobacter *	100
**41**	3	0	Unc. *Geobacter* sp.	EU266817	99.76	Deltaproteobacteria	*Geobacter *	100
**42**	2	0	Unc. *Geobacter* sp.	EU266841	99.16	Deltaproteobacteria	*Geobacter *	100
**43**	5	0	*Geobacter lovleyi *	NR_074979	99.03	Deltaproteobacteria	*Geobacter *	100
**44**	1	0	*Geobacter thiogenes *	NR_028775	97.48	Deltaproteobacteria	*Geobacter *	100
**45**	1	1	Unc. Firmicutes bacterium	HQ003641	98.70	Clostridia	Clostridiales Incertae Sedis XII	100
**46**	0	20	Unc. Firmicutes bacterium	HQ003641	99.45	Clostridia	*Acidaminobacter *	86
**47**	0	1	Unc.* Clostridium* sp.	FM204998	100.0	Clostridia	*Clostridium XlVa *	100
**48**	0	3	*Acetobacterium malicum *	NR_026326	99.53	Clostridia	*Acetobacterium *	100
**49**	1	0	Unc. bacterium	AB759668	95.24	Bacteria	Firmicutes	100
**50**	2	0	Unc. rumen bacterium	AB615047	94.24	Lentisphaerae	*Victivallis *	97
**51**	2	0	Unc. *Cytophaga* sp.	EU809766	99.35	Lentisphaerae	*Victivallis *	97
**52**	1	0	Denitrifying bacterium	FJ802233	98.54	Ignavibacteria	*Ignavibacterium *	91
**53**	0	8	Unc. *Bacteroides* sp.	FJ862827	99.18	Bacteroidia	*Parabacteroides *	100
**54**	0	3	*Macellibacteroides fermentans *	NR_117913	99.08	Bacteroidia	*Parabacteroides *	99
**55**	0	1	Unc. Bacteroidetes bacterium	FJ535139	94.64	Bacteroidia	Porphyromonadaceae	88
**56**	0	1	Unc. Bacteroidetes bacterium	DQ676360	99.30	Bacteroidia	Bacteroidales	99
**57**	1	0	Unc. *Prolixibacter* sp.	JQ723616	97.85	Bacteria	Bacteroidetes	100
**58**	1	0	Unc. *Geobacter* sp.	JQ086897	98.72	Deltaproteobacteria	*Geobacter *	100
**59**	1	0	*Geobacter lovleyi *	NR_074979	99.37	Deltaproteobacteria	*Geobacter *	100
**60**	1	0	*Sulfuricurvum kujiense *	NR_074398	99.29	Epsilonproteobacteria	*Sulfuricurvum *	100

^A^Confidence threshold of the RDPII CLASSIFIER Tool is 80%.

## References

[1] Stringer R., Johnston P. (2001). Chlorine and the environment: an overview of the chlorine industry. *Environmental Science and Pollution Research*.

[2] Ruder A. M. (2006). Potential health effects of occupational chlorinated solvent exposure. *Annals of the New York Academy of Sciences*.

[3] Hughes K., Meek M. E., Caldwell I. (1994). 1,2-Dichloroethane: evaluation of risks to health from environmental exposure in Canada. *Journal of Environmental Science and Health*.

[4] Smidt H., de Vos W. M. (2004). Anaerobic microbial dehalogenation. *Annual Review of Microbiology*.

[5] Field J. A., Sierra-Alvarez R. (2004). Biodegradability of chlorinated solvents and related chlorinated aliphatic compounds. *Reviews in Environmental Science and Bio/Technology*.

[6] Macbeth T. W., Cummings D. E., Spring S., Petzke L. M., Sorenson K. S. (2004). Molecular characterization of a dechlorinating community resulting from in situ biostimulation in a trichloroethene-contaminated deep, fractured basalt aquifer and comparison to a derivative laboratory culture. *Applied and Environmental Microbiology*.

[7] Marzorati M., Borin S., Brusetti L. (2006). Response of 1,2-dichloroethane-adapted microbial communities to *ex-situ* biostimulation of polluted groundwater. *Biodegradation*.

[8] Hirschorn S. K., Grostern A., Lacrampe-Couloume G. (2007). Quantification of biotransformation of chlorinated hydrocarbons in a biostimulation study: Added value via stable carbon isotope analysis. *Journal of Contaminant Hydrology*.

[9] Marzorati M., Balloi A., de Ferra F. (2010). Bacterial diversity and reductive dehalogenase redundancy in a 1,2-dichloroethane-degrading bacterial consortium enriched from a contaminated aquifer. *Microbial Cell Factories*.

[10] Arjoon A., Olaniran A. O., Pillay B. (2013). Enhanced 1,2-dichloroethane degradation in heavy metal co-contaminated wastewater undergoing biostimulation and bioaugmentation. *Chemosphere*.

[11] Holliger C., Wohlfarth G., Diekert G. (1999). Reductive dechlorination in the energy metabolism of anaerobic bacteria. *FEMS Microbiology Reviews*.

[12] Maillard J., Schumacher W., Vazquez F., Regeard C., Hagen W. R., Holliger C. (2003). Characterization of the corrinoid iron-sulfur protein tetrachloroethene reductive dehalogenase of *Dehalobacter restrictus*. *Applied and Environmental Microbiology*.

[13] van de Pas B. A., Smidt H., Hagen W. R. (1999). Purification and molecular characterization of ortho-chlorophenol reductive dehalogenase, a key enzyme of halorespiration in *Desulfitobacterium dehalogenans*. *Journal of Biological Chemistry*.

[14] Neumann A., Siebert A., Trescher T., Reinhardt S., Wohlfarth G., Diekert G. (2002). Tetrachloroethene reductive dehalogenase of *Dehalospirillum multivorans*: substrate specificity of the native enzyme and its corrinoid cofactor. *Archives of Microbiology*.

[15] Grostern A., Edwards E. A. (2009). A characterization of a *Dehalobacter* coculture that dechlorinates 1,2-dichloroethane to ethene and identification of the putative reductive dehalogenase gene. *Applied and Environmental Microbiology*.

[16] Marzorati M., de Ferra F., van Raemdonck H. (2007). A novel reductive dehalogenase, identified in a contaminated groundwater enrichment culture and in *Desulfitobacterium dichloroeliminans* strain DCA1, is linked to dehalogenation of 1,2-dichloroethane. *Applied and Environmental Microbiology*.

[17] Maymó-Gatell X., Anguish T., Zinder S. H. (1999). Reductive dechlorination of chlorinated ethenes and 1,2-dichloroethane by ‘Dehalococcoides ethenogenes’ 195. *Applied and Environmental Microbiology*.

[18] He J., Ritalahti K. M., Yang K.-U., Koenigsberg S. S., Löffler F. E. (2003). Detoxification of vinyl chloride to ethene coupled to growth of an anaerobic bacterium. *Nature*.

[19] Moe W. M., Yan J., Nobre M. F., da Costa M. S., Rainey F. A. (2009). *Dehalogenimonas lykanthroporepellens* gen. nov., sp. nov., a reductively dehalogenating bacterium isolated from chlorinated solvent-contaminated groundwater. *International Journal of Systematic and Evolutionary Microbiology*.

[20] Bowman K. S., Nobre M. F., da Costa M. S., Rainey F. A., Moe W. M. (2013). *Dehalogenimonas alkenigignens* sp. nov., a chlorinated-alkane-dehalogenating bacterium isolated from groundwater. *International Journal of Systematic and Evolutionary Microbiology*.

[21] Maness A. D., Bowman K. S., Yan J., Rainey F. A., Moe W. M. (2012). *Dehalogenimonas* spp. can reductively dehalogenate high concentrations of 1,2-dichloroethane, 1,2-dichloropropane, and 1,1,2-trichloroethane. *AMB Express*.

[22] De Wildeman S., Diekert G., van Langenhove H., Verstraete W. (2003). Stereoselective microbial dehalorespiration with vicinal dichlorinated alkanes. *Applied and Environmental Microbiology*.

[23] Murray A. E., Preston C. M., Massana R. (1998). Seasonal and spatial variability of bacterial and archaeal assemblages in the coastal waters near Anvers Island, Antarctica. *Applied and Environmental Microbiology*.

[24] Wen D., Bai Y., Shi Q. (2012). Bacterial diversity in the polluted water of the Dianchi Lakeshore in China. *Annals of Microbiology*.

[25] van der Wielen P. W. J. J., Bolhuis H., Borin S. (2005). The enigma of prokaryotic life in deep hypersaline anoxic basins. *Science*.

[26] Cytryn E., Minz D., Oremland R. S., Cohen Y. (2000). Distribution and diversity of archaea corresponding to the limnological cycle of a hypersaline stratified lake (Solar Lake, Sinai, Egypt). *Applied and Environmental Microbiology*.

[27] Wright E. S., Yilmaz L. S., Noguera D. R. (2012). DECIPHER, a search-based approach to chimera identification for 16S rRNA sequences. *Applied and Environmental Microbiology*.

[28] Schloss P. D., Westcott S. L., Ryabin T. (2009). Introducing mothur: open-source, platform-independent, community-supported software for describing and comparing microbial communities. *Applied and Environmental Microbiology*.

[29] Hammer Ø., Harper D. A. T., Ryan P. D. (2001). PAST: paleontological statistics software package for education and data analysis. *Palaeontologia Electronica*.

[30] Altschul S. F., Gish W., Miller W., Myers E. W., Lipman D. J. (1990). Basic local alignment search tool. *Journal of Molecular Biology*.

[31] Wang Q., Garrity G. M., Tiedje J. M., Cole J. R. (2007). Naïve Bayesian classifier for rapid assignment of rRNA sequences into the new bacterial taxonomy. *Applied and Environmental Microbiology*.

[32] Lorenz M. O. (1905). Methods of measuring concentration of wealth. *Journal of the American Statistical Association*.

[33] Marzorati M., Wittebolle L., Boon N., Daffonchio D., Verstraete W. (2008). How to get more out of molecular fingerprints: practical tools for microbial ecology. *Environmental Microbiology*.

[34] Merlino G., Rizzi A., Schievano A. (2013). Microbial community structure and dynamics in two-stage vs single-stage thermophilic anaerobic digestion of mixed swine slurry and market bio-waste. *Water Research*.

[35] Tamura K., Peterson D., Peterson N., Stecher G., Nei M., Kumar S. (2011). MEGA5: molecular evolutionary genetics analysis using maximum likelihood, evolutionary distance, and maximum parsimony methods. *Molecular Biology and Evolution*.

[36] Regeard C., Maillard J., Holliger C. (2004). Development of degenerate and specific PCR primers for the detection and isolation of known and putative chloroethene reductive dehalogenase genes. *Journal of Microbiological Methods*.

[37] Lovley D. R., Holmes D. E., Nevin K. P. (2004). Dissimilatory Fe(III) and Mn(IV) reduction. *Advances in Microbial Physiology*.

[38] de Wever H., Cole J. R., Fettig M. R., Hogan D. A., Tiedje J. M. (2000). Reductive dehalogenation of trichloroacetic acid by *Trichlorobacter thiogenes* gen. nov., sp. nov.. *Applied and Environmental Microbiology*.

[39] Sung Y., Fletcher K. E., Ritalahti K. M. (2006). *Geobacter lovleyi* sp. nov. strain SZ, a novel metal-reducing and tetrachloroethene-dechlorinating bacterium. *Applied and Environmental Microbiology*.

[40] Kodama Y., Watanabe K. (2004). *Sulfuricurvum kujiense* gen. nov., sp. nov., a facultatively anaerobic, chemolithoautotrophic, sulfur-oxidizing bacterium isolated from an underground crude-oil storage cavity. *International Journal of Systematic and Evolutionary Microbiology*.

[41] Liang S. H., Liu J. K., Lee K. H., Kuo Y. C., Kao C. M. (2011). Use of specific gene analysis to assess the effectiveness of surfactant-enhanced trichloroethylene cometabolism. *Journal of Hazardous Materials*.

[42] Lambo A. J., Patel T. R. (2006). Isolation and characterization of a biphenyl-utilizing psychrotrophic bacterium, *Hydrogenophaga taeniospiralis* IA3-A, that cometabolize dichlorobiphenyls and polychlorinated biphenyl congeners in Aroclor 1221. *Journal of Basic Microbiology*.

[43] Zhao H.-P., van Ginkel S., Tang Y., Kang D.-W., Rittmann B., Krajmalnik-Brown R. (2011). Interactions between perchlorate and nitrate reductions in the biofilm of a hydrogen-based membrane biofilm reactor. *Environmental Science & Technology*.

[44] Narihiro T., Kaiya S., Futamata H., Hiraishi A. (2010). Removal of polychlorinated dioxins by semi-aerobic fed-batch composting with biostimulation of '*Dehalococcoides*'. *Journal of Bioscience and Bioengineering*.

[45] Suyama A., Yamashita M., Yoshino S., Furukawa K. (2002). Molecular characterization of the *PceA* reductive dehalogenase of *Desulfitobacterium* sp. strain Y51. *Journal of Bacteriology*.

[46] Maillard J., Regeard C., Holliger C. (2005). Isolation and characterization of Tn-Dha1, a transposon containing the tetrachloroethene reductive dehalogenase of *Desulfitobacterium hafniense* strain TCE1. *Environmental Microbiology*.

